# *Rhyparida foaensis* (Jolivet, Verma & Mille, 2007), comb. n. (Coleoptera, Chrysomelidae) and implications for the colonization of New Caledonia

**DOI:** 10.3897/zookeys.157.1320

**Published:** 2011-12-21

**Authors:** Jesús Gómez-Zurita

**Affiliations:** 1 Institut de Biologia Evolutiva (CSIC-UPF), Natural Resources-CSIC, Pg. Marítim de la Barceloneta 37, 08003 Barcelona, Spain

**Keywords:** *Rhyparida*, *Dematochroma*, New Caledonia, island disharmony, new combination

## Abstract

The study of external morphology of the New Caledonian leaf beetle *Dematochroma foaensis* Jolivet, Verma & Mille (Chrysomelidae, Eumolpinae, Colaspoidini) substantiates its new combination into the genus *Rhyparida* Baly (Chrysomelidae, Eumolpinae, Nodinini). The species is redescribed here to highlight characters important for suprageneric diagnosis. This is the second species of Nodinini found in New Caledonia, otherwise rich in species of Colaspoidini, raising questions about the paucity of *Rhyparida* and this tribe in New Caledonian fauna, when they are dominant in surrounding archipelagoes, and very rich in potential source areas such as Australia and New Guinea. Some alternative explanations for this pattern are advanced, serving as alternative hypotheses until our knowledge on the ecology of these species improves or supported phylogenetic scenarios become available for this group.

## Introduction

**Figure 1.**
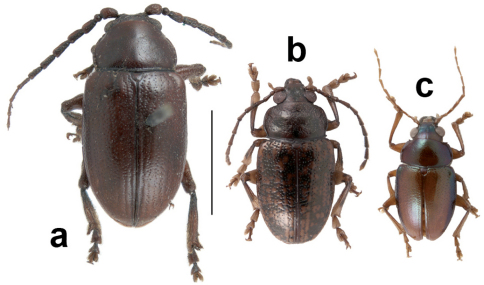
Habitus of three species of *Dematochroma* **a** Male holotype of *Dematochroma piceum* Baly from Lord Howe Island (N.H.M., London) **b** male of *Dematochroma laboulbenei* (Montrouzier) from Thio, New Caledonia (voucher no. IBE-JGZ-NC-0112; I.B.E., Barcelona), and **c** male of *Dematochroma antipodum* (Fauvel) from L’Aoupinié, New Caledonia (voucher no. IBE-JGZ-NC-0144; I.B.E., Barcelona). Scale bar = 5 mm.

Generic attributions of New Caledonian Eumolpinae are currently in need of revision. Montrouzier (1861) and Fauvel (1862) described two medium sized species of Eumolpinae from the archipelago as *Edusa laboulbenei* Montrouzier and *Chalcoplacis antipodum* Fauvel, respectively. Chapuis (1874) described another New Caledonian species within his “Colaspitae” and under a new genus, *Thasycles cordiformis* Chapuis, which was later synonymised with Montrouzier’s taxon (Lefèvre 1876). Finally, Lefèvre (1885) ranked the two recognized New Caledonian taxa into the genus *Dematochroma* Baly, characterized by the species *Dematochroma picea* Baly, 1864, an endemic eumolpine from Lord Howe island in the so-called Lord Howe Rise, a marine ridge separated from the Norfolk Ridge, the oceanic feature where New Caledonia belongs to (Keast 1996). The three species have markedly divergent external appearance, perhaps as much as to be treated as different genera (Fig. 1). Heller (1916) acknowledged the differences between the forms from New Caledonia and Lord Howe, highlighting the insufficient justification by Lefèvre (1885) to place them together, and preferred to treat them in different genera—against the choice of Clavareau (1914) — maintaining Chapuis’ name *Thasycles* for the Neocaledonian taxa. In fact, he described under *Thasycles* six new species of Eumolpinae, again markedly divergent among each other and from either previously described taxon (see also Gómez-Zurita in press). In the absence of explicit diagnostic characters, his decision to rank species so different under the same generic name was mostly based on the relatively large size of these species and perhaps the prejudice of a fauna evolved in isolation from one or at most few ancestors. In the same tradition, Pierre Jolivet and his co-authors (Jolivet et al. 2007a, b, c, 2009) described many New Caledonian eumolpine beetles, recovering the generic name *Dematochroma*, whereby the distinguishing feature to place the new species under this genusis mainly their moderate size (5–9 mm long; Jolivet et al. 2007b).

Size as a systematic criterion is liable to taxonomic confusion. In my initial steps to understand the systematic structure of New Caledonian Eumolpinae above the species level, both using morphological and DNA-based criteria, stood out one example in need of additional study. The 6 mm long species described as *Dematochroma foaensis* Jolivet, Verma & Mille, 2007a: 43 belongs into a distantly related suprageneric rank compared to *Dematochroma* or most other New Caledonian Eumolpinae. Indeed, after *Stethotes bertiae* Jolivet, Verma & Mille, 2007b: 81 it is the second representative reported from this archipelago as belonging into the tribe Nodinini, as opposed to Colaspoidini, where *Dematochroma* and most other New Caledonian species appear to belong. The species shows highly divergent characters as compared to *Dematochroma sensu auctorum* or any other Eumolpinae in New Caledonia. These include the lack of dorsal longitudinal groove on pygidium, meso- and metatibiae with preapical emargination, and bifid claws. A closer analysis of morphology of several specimens showed it to present the characters considered by previous authors to diagnose the genus *Rhyparida* Baly, 1861 (e.g., Gressitt 1969). Thus, herein, I propose the name *Rhyparida foaensis* (Jolivet, Verma & Mille), comb. n. The original diagnosis for the species was succinct and lacked mention to those systematic characters important for the recognition of the species and its correct placement in the system of Eumolpinae. Thus, a redescription is provided below, with illustrations of male and female genitalia for the first time, as well as a discussion about the presence of this isolated Nodinini in New Caledonia.

## Taxonomy

### Redescription of Rhyparida foaensis (Jolivet, Verma & Mille)

#### Rhyparida foaensis

(Jolivet, Verma & Mille)

http://species-id.net/wiki/Rhyparida_foaensis

##### Material examined.

Type material: (1) Holotype, one male, La Foa, 21°44S, 165°54E, 10 February 2004, M’bouéri R. M. leg. (Museum National d’Histoire Naturelle, Paris); (2) Paratype, one female, Ouégoa, Mandjélia, 20.39683°S, 164.53218°E, 787m, 7–8 February 2005, S. Cazères & C. Mille leg. (Museum National d’Histoire Naturelle, Paris). Other material: (3) two females, Caavatch (=Kaavac), 5 February 1977, Dr. J. Balogh leg. (Hungarian Natural History Museum, Budapest); (5) three females, Province Sud, Camp Brun, 14 March 1994, on *Melaleuca quinquenervia*, M. Schöller leg. (M. Schöller coll., Berlin); (4) one female, Province Nord, Hienghene 20.69545°S, 164.94274°E, 24m, 8 April 2008, J. Gómez-Zurita leg. (J. Gómez-Zurita coll., voucher no. NC-0110, Institute of Evolutionary Biology, Barcelona).

##### Description.

**Figure 2.**
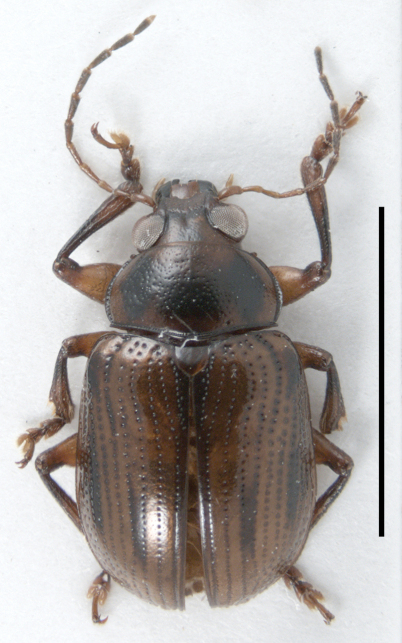
Habitus of *Rhyparida foaensis* (Jolivet, Verma & Mille). Scale bar = 5 mm.

*Habitus* (Fig. 2). Body stout, elongated oval (6.1 mm long, 3.4 mm wide), moderately convex. Ground color orange testaceous, with infuscate head sutures, inverted triangle on frons, apical antennal segments, margins and discal markings on pronotum, scutellum, elytral suture, humeri, medially for short distance on third and seventh elytral intervals, apex of femora, basal half of tibiae, episterna and ventral thoracic segments; mandibles black.

Head large, deeply inserted into pronotum, nearly to upper eye margin; surface very delicately microreticulated; vertex weakly convex, very finely, rather densely and homogeneously punctured, with very fine median longitudinal impression, becoming progressively larger, on depressed longitudinal area on frons, joined apically to transversally widely obtuse fronto-clypeal suture. Clypeus wider than long, subtrapezoidal, depressed apically, with deep median semicircular apical emargination, flanked laterally by shortly produced denticles; surface microreticulated, with larger, deeper punctures than those on vertex, bearing minute, very fine setae anteriorly. Labrum as long as wide; surface finely microreticulated; sides feebly convergent towards round anterior angles; apex depressed and weakly emarginated; anterior angles with one pair of nearly adjacent fine golden setae; two setae anteriorly on disc. Genae very short, with some fine setae below eye margin. Eyes very big, dorsoventrally elongated; deeply emarginated at inner border for antennal insertion; supraocular margin furrowed, furrow not surpassing eye margin above, with long, yellowish dorsal seta. Space for antennal insertion concave, slightly raised dorsally above clypeus level; microreticulated, unpunctured, with one anterior, oblique fine golden seta. Antennae long and slender, reaching basal third of elytra; scape long, weakly flattened and arched antero-posteriorly; second antennomere elongated, slightly clavate, weakly curved, 0.66x as long as first; third segment straight, as long as second; antennomeres 4–5 subcylindrical, slightly shorter than scape, narrow and slender; 6–10 as long as scape, slightly widened towards apex, densely setose; apical antennomere longest, sharply pointed and paler at apex. Maxillary palpi short, slender; apical palpomere elongated, subconical.

Pronotum transverse, 0.58× as long as wide between posterior angles, shorter than head, transversally convex, especially at anterior angles; posterior border weakly bisinuated with weakly projecting median lobe, finely margined with premarginal line of dense dot-like impressions; posterior angles laterally projecting as small teeth continuing basal margin, with large apical setigerous pore; anterior border nearly straight, finely margined at sides, with margin broader and more imprecisely defined at middle; anterior angles laterally and slightly obliquely projecting as small teeth with large setigerous pore at apex; sides broadly curved, wider behind middle; lateral margins relatively wide, flat, glossy, with internal row of dense round impressions; pronotal surface delicately microreticulated, rather uniformly and densely covered by shallow, moderate punctures, smaller, almost disappearing near borders. Anterior border of hypomeron more or less straight, regularly continuing profile of anterior border of pronotum with that of prosternum, both remaining largely separated by anterior margin of hypomeron (see Fig. 4f in Gressitt 1967); hypomera finely alutaceous, unpunctured, with shallow, wavy longitudinal impressions on disc; posterior border of hypomera surrounding procoxae posteriorly for 2/3 of their width, joining apex of prosternal process laterally, enclosing procoxal cavities behind. Prosternum narrow, slightly convex before coxae; anterior border with slightly raised broad margin and weakly emarginated medially; very finely alutaceous, with scattered, fine long yellowish setae; prosternal process broad, as wide as base of femora between coxae, progressively widening apically, following contour of coxae to join posterior border of hypomera; apex of prosternal process straight, twice as wide as width between coxae. Procoxae ovoid, slightly transverse. Combined mesanepisternum and mesepimeron subtrapezoidal, transverse, finely alutaceous, unpunctured. Mesoventrite relatively long, glossy, unpunctured; process long, spatula-like, apex convex, glossy, with few scattered very fine yellowish setae. Metanepisterna long, finely microreticulated, with scattered minute punctures and very fine, short recumbent whitish setae. Metaventrite as long as first abdominal ventrite; disc below level of mesosternal process, glossy, nearly unpunctured; sides finely alutaceous, with scattered minute punctures and very fine, short whitish setae; posterior border with short median notch.

Scutellum as long as broad at base, sides straight, weakly divergent at basal 2/3, curved at obtuse angle to obtusely pointed apex; surface finely alutaceous, unpunctured. Elytra slightly broader than base of pronotum; humeri round, slightly callose; sides very feebly curved, with maximum width behind middle, and regularly curved to broadly round apex; margins feebly explanate, entirely visible from above; surface shiny, with dense unordered minute punctures and regular series of strong punctures separated at most by distance equal to their diameter; short scutellar striae of some 14 punctures starting before middle of scutellum and obliquely directed to suture; sutural striae reaching from base of elytra to sutural angles, joining marginal striae at inner edge of explanate margin of elytra; four longitudinal discal striae from base of elytra joining successively to apical ends of ninth, eighth, seventh and sixth striae on preapical declivity of elytra; basal ends of striae 6–8 behind humeri and of premarginal stria 9 behind middle of elytra; short premarginal posthumeral striae, curved and convergent with elytral margin before middle of elytra; space between striae 7 and 8, medially and at lateral declivity of elytra occupied by two additional shorter longitudinal striae convergent at both ends; darkened sutural interval, humeri, elongated spots medially on disc on third interval and more advanced at lateral declivity of elytra on seventh interval between stria 7 and internal row of additional posthumeral striae. Epipleura flat, unpunctured, shiny, broad basally and gradually narrowing toward apex; only visible laterally below humeri. Species fully winged.

Profemora spindle-shaped at basal 3/4, nearly cylindrical at apical quarter; extremely finely alutaceous with scattered minute punctures and very short appressed setae on basal 3/4 and coarser punctures and longer setae at apical 1/4. Protibiae very slightly curved inward, gradually widened toward apex; with several fine longitudinal ridges and longitudinal series of semierect golden setae at intervals; apex concave, obliquely cut for tarsal insertion, densely setose internally. Protarsi 0.6× as long as protibiae; first tarsomere slightly expanded laterally, longer than wide at concave apex; second shorter than first, triangular with broadly concave apex; third deeply and narrowly bilobed; fifth longer than tarsomeres 2–3, slender, subparallel, ventrally curved; claws bifid, weakly divergent, long, sharp, with short, sharp inner teeth. Median and hind legs very similar to anterior legs, but tibiae straight, with conspicuous preapical emargination externally, margined by fringe of erect golden setae and apex not densely setose internally. Abdominal ventrites finely microsculptured, shiny, narrow, strongly transverse, with posterior border increasing concavity from ventrites one to four, finely but more or less uniformly punctured and with very fine, short whitish setae; sides corrugated; anterior process between metacoxae of first abdominal ventrite broader than long, regularly curved; last abdominal ventrite very feebly emarginated.

**Figure 3.**
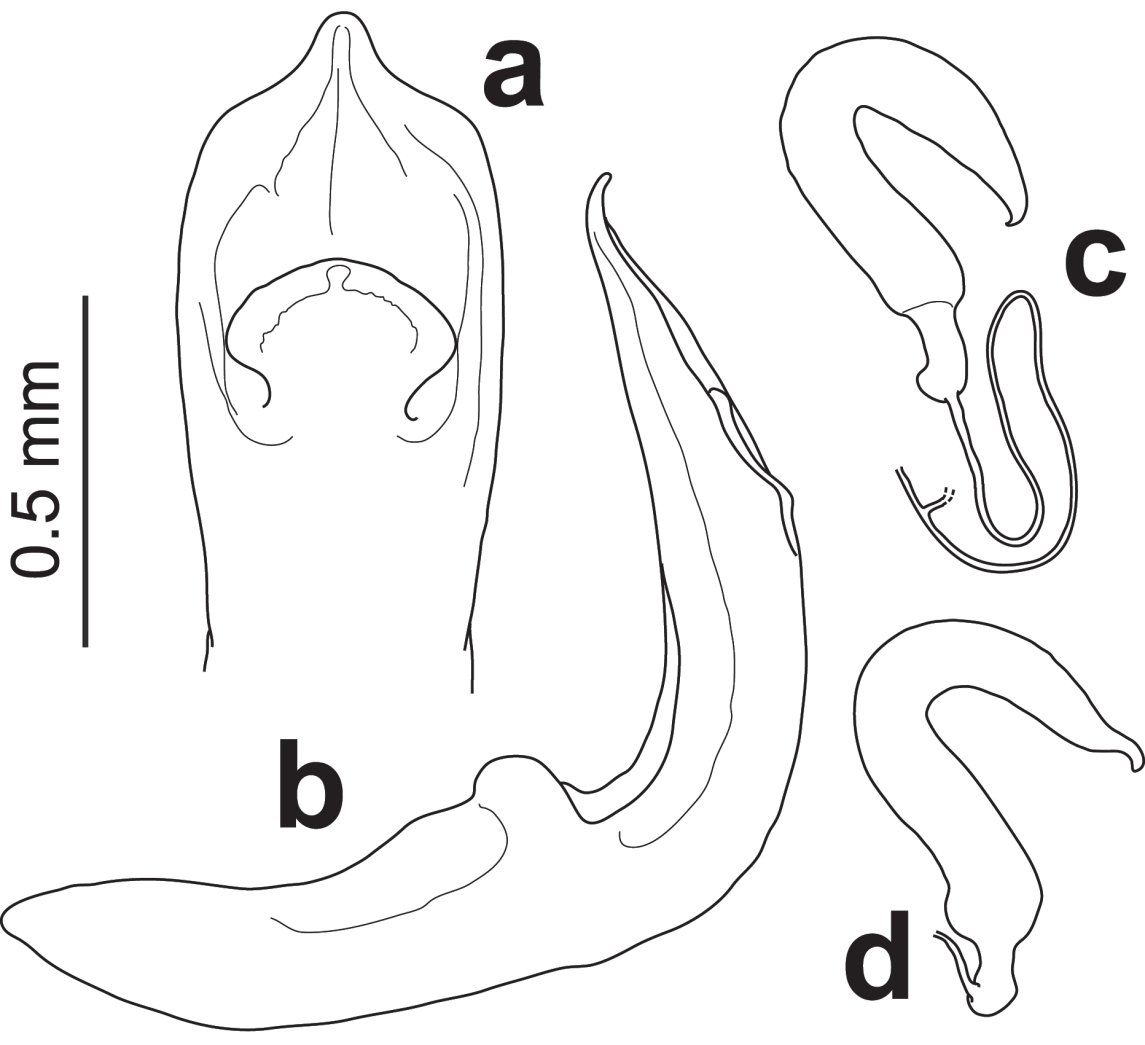
Male (**a** dorsal **b** lateral) and female **c, d** genitalia of *Rhyparida foaensis* (Jolivet, Verma & Mille).

Median lobe of the aedeagus (Fig. 3a, b) strongly bent at right angle near base, dorso-ventrally flattened and nearly straight at apical 2/3; sides slightly divergent, reaching maximum width at mid-level of ostium, feebly converging before abruptly tapering at obtuse angle before apex; apex anteriorly prolonged as blunt median triangular denticle curved dorsally; median dorsal flap broad, spatula-like, with short narrow base 0.5× as wide as broadest point medially, before regularly curved nearly semicircular apex. Spermatheca (Fig. 3c, d) U-shaped with pump slightly shorter than receptacle, gradually narrowing towards curved pointed apex; proximal end slightly broadened before narrow elongated basal appendix attached prebasally to very fine, transparent spermathecal duct; spermathecal gland apparently attached to spermathecal duct distally from spermatheca at 1.5× its length.

### Diversity and distribution of Rhyparida

**Figure 4.**
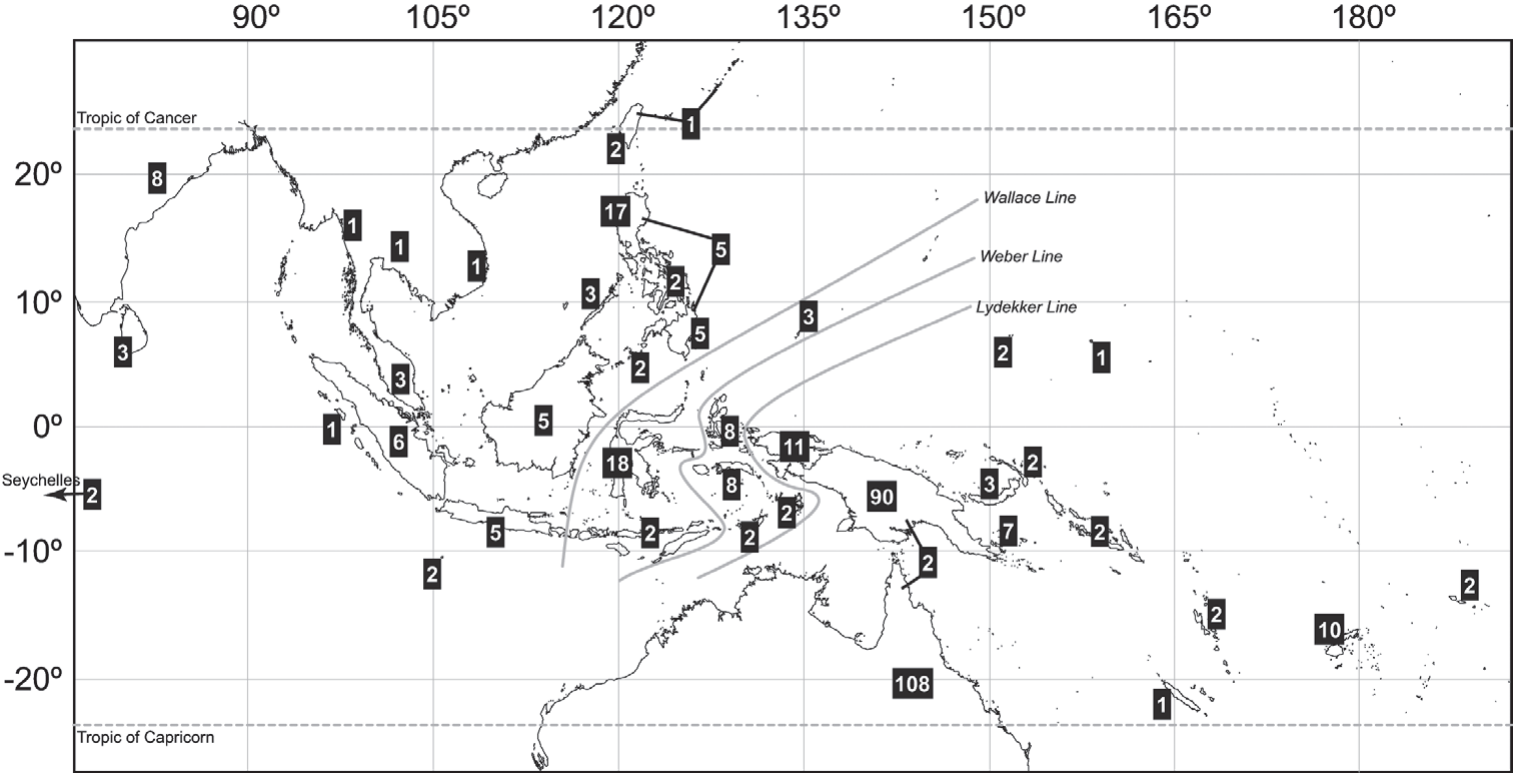
Distribution and diversity of *Rhyparida* Baly species worldwide.

As it occurs with most Eumolpinae genera, the objective limits of *Rhyparida* need to be revised and it is possible that profound changes will affect the systematics of the group (C.A.M. Reid, Australian Museum, pers. comm.). However, before this revision is attempted, following the latest treatments of the genus by several specialists, it is possible to draw some preliminary conclusions about the diversity and biogeography of the genus. Clavareau’s (1914) catalogue lists 166 species of *Rhyparida*, an increase of 34.3% over the account by Lefèvre (1885), thirty years earlier. Today, there are 361 species recognized as belonging into the genus *Rhyparida*, which appear predominantly distributed in Australia (110 species) and the main island of New Guinea (99 species). The remaining species are mostly distributed in the Philippines (32 species), Sulawesi (18 species) and many other islands of Indonesia, as well as in several archipelagoes of the Micronesia and Melanesia (Fig. 4). Very few species occur in continental South East Asia. Interestingly, the genus had not been reported so far from New Caledonia, despite all other surrounding archipelagoes having several species, including Fiji with ten recognized taxa (Bryant and Gressitt 1957), and that the genus reaches as far east as Samoa (Gressitt 1957).

It is largely elusive understanding why such a diverse genus like *Rhyparida* is so rare in New Caledonia, considering the old age of the island, its relatively large size, its ecological diversity and its relative proximity to species-rich source areas such as New Guinea and Australia, as compared to Fiji, for instance, comparatively rich in species of *Rhyparida*. Island disharmony is a well-known biogeographic pattern, and very common in the case of insects in Pacific islands (see Gillespie and Roderick 2002). Thus, *Rhyparida* could represent one more example of biased composition of an island community. But perhaps the attention should be on Fiji and a disharmonic excess of Nodinini, not only *Rhyparida* but several other genera as well, compared to surrounding archipelagoes (Bryant and Gressitt 1957). Fiji supports in turn a comparatively poor Colaspoidini fauna, highly diverse in New Caledonia. In any case, in the absence of a reconstruction for the evolutionary history of this group, whatever explanation we attempt at these patterns remains speculative. Chance determines that island biotas are a non-representative sample of their continental counterparts, and the classical mechanistic justification of differential odds for initial colonization of an island invokes dispersal capabilities of the species in potential sources (Grant 1998). We do not have any reason to believe *a priori* that *Rhyparida* is less suited for transoceanic dispersal compared to other eumolpines such as *Dematochroma*, which have reached, successfully colonized and radiated in New Caledonia. For instance, all New Caledonian eumolpines, including *Rhyparida foaensis*, are winged, the same as their continental relatives. And of course, the presence of the genus in Samoa argues against inherent limitations to dispersal potential.

If differences in ability for dispersal compared to other eumolpines are not obvious, another possibility is that successfully colonizing *Rhyparida* (or other Nodinini for that matter) were outcompeted by local stable populations of Colaspoidini, in this case. Again, and considering the generally eclectic ecologies of these animals, their notable success in similar geographic scenarios also rich in other eumolpines, and the diversity of suitable habitats offered by New Caledonian ecosystems, it is difficult to admit that such a fierce antagonism and exclusion can affect settlement chances for representatives of an entire beetle tribe.

Yet another possibility is that ecological requirements for Nodinini, or *Rhyparida* in particular, are actually stricter than considered *a priori*, and not available in New Caledonia, compared to the mainland or surrounding oceanic islands. This hypothesis could be evaluated examining for instance the association of *Rhyparida* species to specific soils, types of vegetation or specific plants throughout its range and confirming the absence (or rarity) of these conditions in New Caledonia, remarkable and quite unique for its geologic and mineral characteristics (Jaffré 1993; Morat 1993). However, perhaps the importance of host plants in this specific case of island disharmony could be neglected, since *Rhyparida* appears in the literature associated to many different hosts, most of them or their relatives present in New Caledonia. Species of *Rhyparida* have been reported as feeding on dicot Anacardiaceae (Sapindales), Asteraceae (Asterales), Dilleniaceae (Dilleniales), Loganiaceae (Gentianales), Malvaceae (Malvales), Moraceae (Rosales) and Rhizophoraceae (Malpighiales), and monocot Arecaceae (Arecales), Pandanaceae (Pandanales), and Poaceae (Poales) (Bryant and Gressitt 1957; Chûjô and Kimoto 1961; Gressitt 1955, 1967), with species like *Rhyparida coriacea* Jacoby and *Rhyparida carolina* Chûjô found and explicitly reported on many hosts (Gressitt 1955, 1967). Indeed, as it occurs with many eumolpines, it is possible that *Rhyparida* species are polyphagous as root feeding larvae, but also as adults (Jolivet and Verma 2002). If this were the case, they would have a high colonization potential of new habitats, particularly those offering such a diverse range of potential hosts as New Caledonia, but also intermediate islands along their possible colonization routes. The host or hosts of *Rhyparida foaensis* are not known, but some of the specimens available for study were collected on the so-called *niaouli*, a dominant shrub in savannah-like environments in the south of Grande Terre currently included in the genus *Melaleuca* (Myrtales: Myrtaceae), very diverse in Australia and with a similar range as the genus *Rhyparida*.

A last possibility about the paucity of Nodinini in New Caledonia and worth consideration here is that there may be several species in the archipelago still awaiting discovery. Considering the intense sampling in the recent past and the conspicuous characters diagnosing this tribe, although it is likely that new species will be discovered, it appears improbable that the catalogue of New Caledonian Nodinini will grow to a number of species comparable to that found in Fiji or even Samoa, the later with at least eight species among *Rhyparida*, *Stethotes* and *Stygnobia* (Gressitt 1957).

The number of questions that this intriguing pattern suggest and the few, speculative answers available, highlight the importance of further research on New Caledonian fauna, from biodiversity and ecological surveys to phylogenetic analyses which will help understanding the history of colonization and diversification on this remote biodiversity hotspot.

### Replacement names for the genus Rhyparida

During the course of this study, several homonyms were detected affecting the genus *Rhyparida* Baly, which need name replacements to avoid ambiguity. *Rhyparida leana* nom. n. (after Arthur Mills Lea) is proposed as replacement name for the Australian species *Rhyparida apicipennis* Lea, 1915, name preoccupied by a species from Fergusson Island (Papua New Guinea) described by Jacoby (1898). Both Lea (1915) and Weise (1922) used the name *Rhyparida pallidula* to describe species from Australia and the Philippines, respectively; the name *Rhyparida weiseana* nom. n. (after Julius Weise) is suggested to replace Weise’s younger taxon. Finally, Lea (1915) named an Australian species using the same name, *Rhyparida prosternalis*, previously proposed by M. Jacoby for a species found in Indonesian Papua (Jacoby 1894); Lea’s name is thus replaced here by *Rhyparida reiterata* nom. nov. (from post-classical Latin *reiteratus* = repeated).

## Supplementary Material

XML Treatment for Rhyparida foaensis
